# Changes in LDL-C levels among primary health care patients with hypertension, coronary artery disease, and diabetes during a 36-month follow-up

**DOI:** 10.1080/02813432.2026.2718269

**Published:** 2026-09-15

**Authors:** Petra Valkonen, Ulla Mikkonen, Hannu Kautiainen, Pekka Mäntyselkä, Nina Tusa

**Affiliations:** a Institute of Public Health and Clinical Nutrition, University of Eastern, Finland; b Teaching Clinic, Wellbeing Services County of North Savo, Kuopio, Finland; c Folkhälsan Research Center, Helsinki, Finland; d Educational Services, Wellbeing Services County of North Savo, Kuopio, Finland

**Keywords:** LDL, cholesterol, dyslipidaemias, primary health care, atherosclerotic cardiovascular disease, hypolipidemic agents

## Abstract

**Background and aims:**

Low-density lipoprotein cholesterol (LDL-C) level is one important modifiable risk factor for cardiovascular diseases. This study aimed to describe the characteristics of primary health care patients with hypertension, coronary artery disease, or diabetes according to their LDL-C levels and to observe changes in these levels and medications during the 36-month follow-up.

**Methods:**

This study was based on a clinical trial, conducted in a health centre in Siilinjärvi, Finland, between 2017 and 2021. A total of 507 patients with hypertension, coronary artery disease, or diabetes were divided into four groups based on their baseline LDL-C levels to align with the dyslipidaemia guideline risk categories as follows: LDL-C (mmol/L) in Group 0 (< 1.4), Group 1 (1.4–1.8), Group 2 (>1.8–2.6), and Group 3 (> 2.6).

**Results:**

The mean age at baseline was 69 years (58% females). All patients in Groups 0 and 1 already used lipid-lowering medication. A lower LDL-C level at baseline was statistically significantly associated with higher age, lower blood pressureand lower alcohol consumption. There were changes in the pharmacologically active substances prescribed in all groups. During the 36-month follow-up, the mean LDL-C values decreased statistically significantly in group 3 (−0.76 mmol/L, 95% CI −0.90 to −0.62, −21%, *p* < .001: [ANCOVA]). The highest increase in lipid-lowering medication prescriptions occurred within this group.

**Conclusions:**

This study provides a perspective on real-word treatment patterns and changes in medications prescribed as the recommendations change over time. During a period of increasing pharmacotherapeutic opportunities, this study, conducted in everyday clinical practice suggests that common lipid-lowering medications (atorvastatin and rosuvastatin) are most frequently used for primary health care patients, and are associated with a significant decrease in LDL-C levels. A greater decrease was observed among patients with higher baseline LDL-C values. Despite decreases in LDL-C, this study suggests that the gap remains between guideline-recommended treatment targets and achieved LDL-C levels.

## Introduction

Primary health care bears the main responsibility for identifying dyslipidaemia and supporting patients’ treatment adherence [1]. Atherosclerotic cardiovascular diseases (ASCVD) are the leading cause of death globally [2]. Dyslipidaemia is a condition in which the lipid profile of plasma is unfavourable, causing an increased risk of development of ASCVDs [3]. According to the Finnish Current Care Guidelines, dyslipidaemia is defined as having a plasma low-density lipoprotein cholesterol (LDL-C) concentration above 3 mmol/L, a plasma triglyceride above 1.7 mmol/L, or high-density lipoprotein cholesterol below 1.0 mmol/L in males or 1.2 mmol/L in females [1]. The most common form of dyslipidaemia is hypercholesterolaemia, which is mainly characterised by increased LDL-C [4], a major ASCVD risk factor that can be modified with appropriate treatment [3,5].

According to current guidelines, there are four different target LDL-C levels with lipid-lowering treatments that are based on the patient’s individual risk of developing ASCVD. The treatment target for LDL-C is < 1.4 mmol/L for particularly high-risk patients, < 1.8 mmol/L for high-risk patients, < 2.6 mmol/L for moderate risk patients, and < 3 mmol/L for the rest of the population (the low-risk patients). The treatment targets for LDL-C have progressively tightened as more research is conducted [1,3,6].

The European Atherosclerotic Society (EAS) and the European Society of Cardiology (ESC) state in their guideline for dyslipidaemia that timely, active secondary prevention in primary health care decreases morbidity [3,5]. The meta-analysis covering 170,000 patients showed a 10% decline in all-cause mortality per LDL-C reduction of 1.0 mmol/L [7]. Therefore, the treatment should be effective and incorporate both drug-free and medication-based approaches [1,3]. The medication-based options include different lipid-lowering medications (LLM), such as statins, ezetimibe, fibrates, bile acid sequestrants, and proprotein convertase subtilisin/kexin type 9 inhibitors (PCSK9-inhibitors). Statins are the first line LLM and are effective both in lowering LDL-C and reducing risk for ASCVD and thus overall-mortality [1,3,5,8].

There seems to be a clinically relevant gap in achieving recommended LDL-C level in general. The da Vinci study, a European-wide cohort study among primary and secondary care patients receiving LLM for primary or secondary prevention found that in 2016 more than half of the patients did not reach the LDL-C treatment target defined by the ESC/EAS [9]. Furthermore, in 2019, over two thirds of those patients did not meet the desired target [9]. When only the secondary prevention patients were selected from the data, the LDL-C level goal attainment was even more unsatisfactory with 80–90% of patients falling short of it in 2019 [9]. In the multinational observational SANTORINI study, another study among primary and secondary care patients at high or very high ASCVD risk receiving LLMs across Europe between 2020 and 2021, showed that 80% of high risk or very high-risk patients did not meet their LDL-C treatment targets [10]. In a 2020 Finnish study among primary health care patients with hypertension and an LDL-C target set below 3 mmol/L, a third of patients using LLM did not meet the treatment target [11]. Despite extensive research on lipid management in general, previous literature provides only little data on adherence to tighter LDL-C targets and LLM use in everyday clinical practice in primary health care.

This study aimed to characterise primary health care patients with hypertension, diabetes, or coronary artery disease according to their LDL-C levels and to assess changes in these levels and LLM prescriptions over a 36-month follow-up.

## Methods

### The study

This analysis is based on a randomised controlled study, Participatory Patient Care Planning in Primary Care (4PHC), conducted at the municipal health centre in Siilinjärvi, Finland, between 2017 and 2021 (ClinicalTrials.gov ID: NCT02992431). In the original study, the patients were randomised into the intervention (participatory care planning process) or usual care group. Based on previously published data on the trial, these two groups did not differ significantly in primary outcome (health-related quality of life or clinical outcomes) at 12 or 36 months [12,13]. Thus, in this study, all patients were analysed as one unified group. Inclusion criteria for the study included age ≥18 years and a physician-diagnosed hypertension (HA), coronary artery disease (CAD), and/or diabetes (DM) based on International Classification of Diseases, 10th Revision (ICD-10). The patients in the HA group did not have CAD or DM. The patients classified into the CAD group could also have HA, and the patients classified into the DM group could have either or both conditions in addition to diabetes. Patients in the terminal phase of any serious condition or in severe cognitive decline were excluded. At baseline, there were 605 patients in total. Of those, 507 were included in this study since they had LDL-C measurements both at baseline and 12 and/or 36 months.

### Outcomes

Main outcome, fasting plasma concentration of LDL-C was collected and analysed in the laboratory of the Kuopio University hospital using common laboratory standards and methods. The patients were divided into four groups based on their baseline LDL-C levels to align with the guideline risk categories as follows: LDL-C under 1.4 mmol/L (Group 0), between 1.4 and 1.8 (Group 1), over 1.8 to 2.6 (Group 2), and over 2.6 (Group 3).

### Other measurements

Glycated haemoglobin A1C (HbA1C) was measured at laboratory after a 12 h fast.

A trained nurse measured both the systolic and diastolic blood pressure after 10 min of resting in a sitting position and height (cm) and weight (kg) in light clothing at baseline at the appointment. The body mass index (BMI) was calculated by dividing the weight (kg) by the height (m^2^) squared from measurements taken by the study nurse at baseline. Data on lipid-lowering medications were retrieved from nurse-verified medication list.

Patients answered a questionnaire concerning sociodemographic factors such as educational background in years and relationship status. Patients’ self-reported additional long-term diseases were asked. Lifestyle factors such as smoking habits (yes or no) and alcohol consumption (the first 2 questions of AUDIT-C [14]) were determined. The level of physical activity was reviewed using the KASARI fit index (Frequency x Intensity x Time [15],) in which the results are scaled from 0 to 100, 100 being the most active.

### Statistical analyses

Descriptive statistics were presented as means with standard deviation (SD) (continuous variables), as medians with interquartile range (IQR) (ordinal variables), or as counts with percentages. The unadjusted hypothesis of linearity across LDL-C levels was tested using the Cochran–Armitage test, linear-by-linear test (categorical variables), or analysis of variance (ANOVA) (continuous variables) with an appropriate contrast. Mean changes between the baseline and 36-month LDL-C measurements were assessed using analysis of variance or covariance (ANCOVA). A possible nonlinear relationship between LDL-C levels and use of LLMs was evaluated using a 3-knot restricted cubic spline logistic model (categorical variables). Normal distributions were evaluated graphically and with the Shapiro–Wilk W test. Stata 18 (StataCorp LP, College Station, TX, USA) were used for the analysis.

## Results

As presented in Table 1, at baseline, 5% of patients had LDL-*C* < 1.4 mmol/L (Group 0), 14% had LDL-C between 1.4 and 1.8 mmol/L (Group 1), 41% had LDL-C between >1.8 and 2.6 mmol/L (Group 2), and 40% had LDL-C over 2.6 mmol/L (Group 3). As seen in Table 1, a lower LDL-C level at baseline was statistically significantly associated with higher age, lower blood pressure, lower alcohol consumption, and fewer self-reported additional long-term diseases.

**Table 1.** Characteristics of the patients at baseline.

	Group 0 LDL-*C* < 1.4 *N* = 25	Group 1 LDL-C 1.4–1.8 *N* = 73	Group 2 LDL-*C* > 1.8–2.6 *N* = 207	Group 3 LDL-*C* > 2.6 *N* = 202	*p* Value
Diagnosis, *n* (%)					<.001^a^ [ χ^2^]
Hypertension	4(16)	14(19)	71(34)	122(60)	
Coronary artery disease	9(36)	20(27)	40(19)	17(8)	
Diabetes	12(48)	39(53)	96(46)	63(31)	
Female, *n* (%)	9(36)	35(48)	109(53)	114(56)	.42^b^ [Cochran–Armitage χ^2^ test]
Age, years, mean (*SD*)	71(9)	71(8)	69(9)	68(9)	.004^b^ [ANCOVA
Body Mass Index (BMI), kg/m^2^, mean (*SD*)	30.7(8.1)	29.4(5.5)	29.5(5.5)	28.8(5.6)	.11^b^ [ANCOVA
Waist, cm, mean (*SD*)					
Women	103(12)	102(16)	97(17)	98(15)	.22^b^ [ANCOVA
Men	105(12)	102(11)	105(11)	104(13)	.87^b^ [ANCOVA
Living with a spouse, *n* (%)	19(76)	48(67)	146(72)	140(69)	.80^b^ [Cochran–Armitage χ^2^ test]
Education, years, mean (*SD*)	9.4(2.9)	10.6(3.2)	10.0(3.0)	10.2(3.1)	.64^b^ [ANCOVA
Smoking, *n* (%)	8(8)	8(12)	3(8)	33(11)	.46^b^ [Cochran–Armitage χ^2^ test]
Alcohol dosages per week, median (IQR)	0.4(0.0,1.0)	0.4(0.0,1.0)	0.8(0.0,1.8)	0.8(0.4,1.8)	.046^b^
Physical activity, Kasari FIT index, mean (*SD*)	47.8(21.9)	41.5(17.8)	40.7(20.8)	41.9(19.2)	.51^b^ [ANCOVA
Blood pressure, mmHg, mean (*SD*)					
Systolic	144(15)	143(17)	145(17)	148(19)	.051^b^ [ANCOVA
Diastolic	79(10)	77(10)	82(10)	84(11)	<.001^b^ [ANCOVA
HbA1c, mmol/mol, mean (*SD*) [number] Patients with diabetes	49(8) 8[12]	44(8) [39]	47(11) [96]	44(10) [63]	.55^b^
Number of self-reported diseases					<.001^b^ [χ^2^ test, linear-by-linear test]
1	6 (24)	6 (8)	42 (20)	59 (29)	
2–3	7 (28)	46 (63)	124 (60)	113 (56)	
>3	12 (48)	21 (29)	41 (20)	30 (15)	

*Note:* LDL-C: low-density lipoprotein cholesterol; HbA1C: glycated haemoglobin A1c.

^a^*p* Value between groups.

^b^*p* Value for linearity of levels between groups.

**Figure 1.**
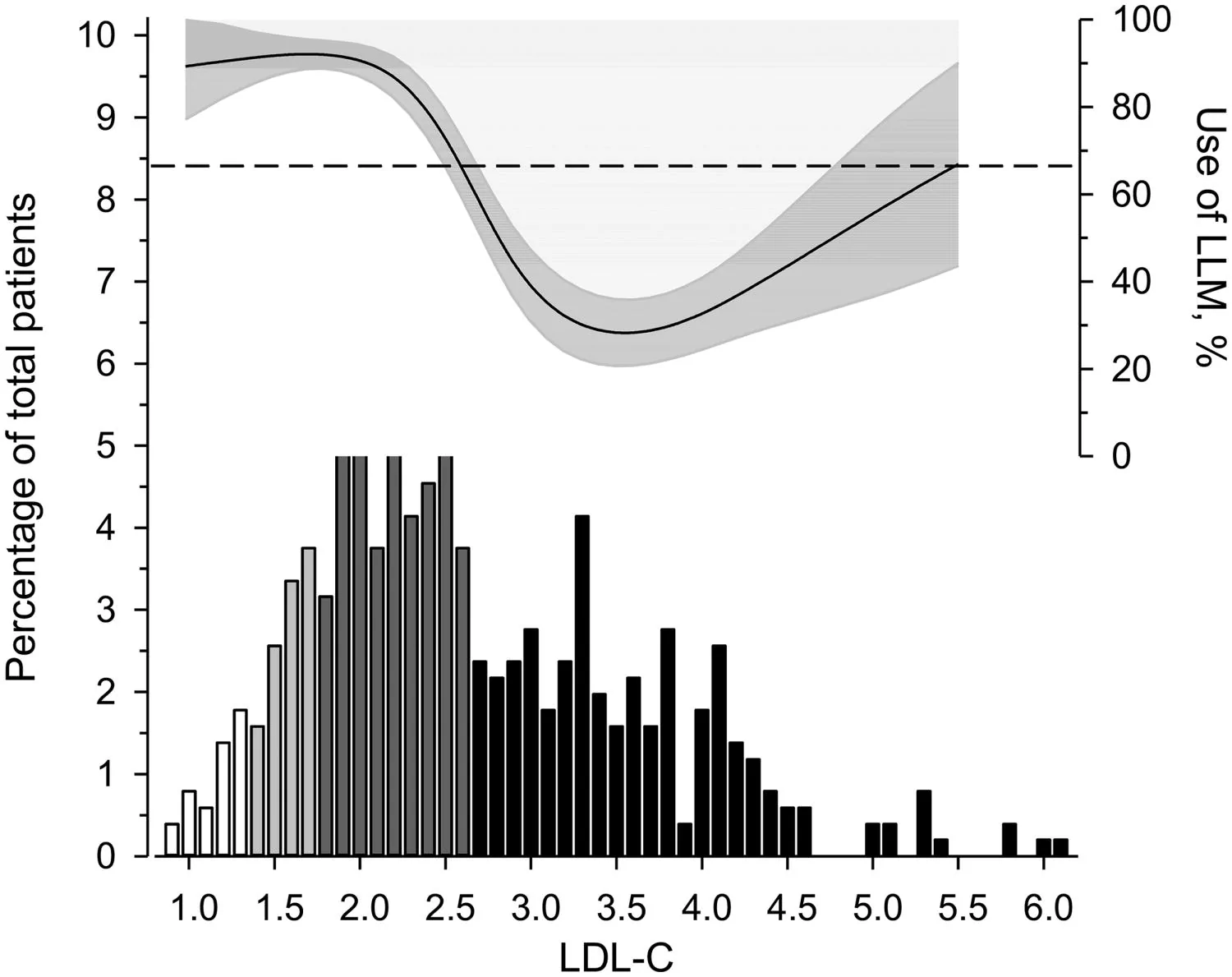
Baseline low-density lipoprotein cholesterol (LDL-C) levels and the percentage of patients prescribed lipid-lowering medications (LLM). The solid line shows the relationship between LDL-C levels and use of LLMs, evaluated using a 3-knot restricted cubic spline logistic model with the grey area representing 95% confidence intervals. The dotted line shows the average medication use. The different shades of grey describe different LDL-C risk groups (0–3) used in the study.

There was a relationship between LLMs and LDL-C levels (Figure 1). The relation was almost a U-shape: The highest use of LLMs was observed in patients with the lowest and highest LDL-C levels.

There was a linear trend in changes in LDL-C levels from baseline to 36 months; the largest decrease being −0.76 in Group 3 (−0.90 to − 0.62, *p* = .001, adjusted for baseline value) as seen in Table 2.

**Table 2.** Baseline LDL-C levels and changes over 36 months by baseline groups.

	Low-density lipoprotein cholesterol at baseline	P for linearity			
	Group 0 <1.4 *N* = 25	Group 1 1.4–1.8 *N* = 73	Group 2 >1.8–2.6 *N* = 207	Group 3 >2.6 *N* = 202	
Baseline	1.17 (0.13)	1.63 (0.13)	2.22 (0.23)	3.60 (0.70)	
After 36 months	1.19 (0.26)	1.58 (0.34)	2.12 (0.65)	2.84 (0.96)	
Change	0.03 (−0.08 to 0.14)	−0.05 (0.13 to 0.03)	−0.10 (−0.19 to 0.01)	−0.76 (−0.90 to −0.62)	0.015* [0.010 baseline adjusted]
Percentage change % (95 % CI)	2 (−2 2 to 27)	−3 (−16 to 9)	−5 (−10 to 0)	−21 (−24 to −19)	

* *p* = .010 after adjustment for baseline value.

Table 3 shows the amount of LLMs prescribed at baseline and at 36 months. At baseline the most commonly prescribed LLM was simvastatin and at 36 months rosuvastatin. At baseline, all patients in Groups 0 and 1 already had prescriptions for LLM. In Group 2 and 3, the percentage of patients prescribed with LLM increased during the 36-month follow-up, with the greatest increase seen in Group 3. Of the patients not using LLM at baseline, 36 (39%) were prescribed LLM during follow-up. Of the patients prescribed with LLM at baseline, 37 (12.3%) did not use LLM after 36 months.

**Table 3.** Percentages of different pharmacologically active substances prescribed, their median dosages, and the percentage of patients with prescriptions, per group at baseline and after 36 months.

	Medications prescribed per group (%)									
	Group 0 LDL-*C* < 1.4		Group 1 LDL-C 1.4–1.8		Group 2 LDL-*C* > 1.8–2.6		Group 3 LDL-*C* > 2.6		Median dose (mg/day)	
ATC^a^ / pharmacologically active substance	Baseline	36 mo	Baseline	36 mo	Baseline	36 mo	Baseline	36 mo	Baseline	36 mo
C10AA01 simvastatin	32.0	28.0	42.5	35.6	32.9	23.7	7.9	5.4	20	20
C10AA05 atorvastatin	16.0	16.0	28.8	24.7	32.9	35.3	13.4	21.3	20	20
C10AA07 rosuvastatin	44.0	40.0	20.5	27.4	11.6	18.4	7.9	21.8	10	10
C10AX09 ezetimibe	8.0	8.0	5.5	6.8	4.3	8.7	5.9	9.9	10	10
C10AA03 pravastatin	0.0	0.0	1.4	1.4	1.0	0.5	3.0	2.0	40	30
C10AA04 fluvastatin	0.0	0.0	1.4	4.1	1.9	1.0	0.5	1.0	80	80
C10AC01 colestyramine	0.0	0.0	1.4	1.4	0.5	0.0	0.5	0.0	600	400
C10BA01 lovastatin and nicotinic acid	0.0	0.0	0.0	0.0	1.0	0.5	1.0	0.5	30^b^	30^b^
C10BA02 simvastatin and ezetimibe	0.0	0.0	0.0	0.0	0.5	0.0	0.5	0.0	30/10	0
C10AB02 bezafibrate	0.0	0,0	0.0	0.0	0.0	0.0	0.5	0.0	400	0
C10AB04 gemfibrozil	0.0	0.0	0.0	0.0	0.0	0.0	0.5	0.5	600	600
C10BX11 atorvastatin. amlodipine and perindopril	0.0	0.0	0.0	1.4	0.0	0.0	0.0	0.5	0	15^c^
C10AX13 evolocumab	0.0	0.0	0.0	0.0	0.0	0.0	0.0	0.5	0	140
C10AX14 alirocumab	0.0	0.0	0.0	0.0	0.0	0.0	0.0	0.5	0	150
Medications prescribed per group (%)	100.0	92.0	100.0	100.0	86.5	87.9	41.6	63.9		

^a^ ATC = Anatomical Therapeutic Chemical.

^b^Dosage refers to lovastatin.

^c^ Dosage refers to atorvastatin.

## Discussion

This study described the changes in low-density lipoprotein cholesterol levels and lipid-lowering medication prescribed over 36 months among primary health care patients with hypertension, coronary artery disease, or diabetes. The LDL-C levels declined statistically significantly during the study period amongst the patients with the highest LDL-C levels at baseline. Regarding LLMs, our results propose that LDL-C levels below 1.8 or 1.4 mmol/L seem to be possible to achieve with statin therapy alone, which is the golden standard for dyslipidaemia treatment.

Despite the evidence of reduced mortality with treatment of hypercholesterolaemia, the treatment targets are rarely achieved [3,9–11,16,17]. CAD and DM patients are at high or very high risk for ASCVD related complications and terminal events. Thus, their individual LDL-C target level is at least below 1.8 or even 1,4 mmol/L. When using this interpretation, 73% of patients in our study in baseline fell short of this presumed target, which is in line with previously referred studies. The Da Vinci study concluded that 80–90% of secondary prevention patients did not meet the desired target in 2019 [9]. Among hypertension patients, one third failed to meet their targets in the Finnish study of 2020 [11]. Still, lowering LDL-C level by just 1 mmol/L can reduce all-cause mortality [3,7,18].

In our study, the majority of patients received statin only, as monotherapy. The most significant change in the LLMs prescribed was a tendency towards switching from simvastatin to rosuvastatin or atorvastatin. The first recommended LLM to be combined with a statin is ezetimibe [1,3,5], but less than 10% of patients in our study were prescribed it at 36 months. In addition, one option for enhancing lipid-lowering treatment is to increase the statin dosage. Our data shows that the three most prescribed statins are being used in low dosages. Only a few patients were prescribed the newer PCSK9-inhibitors. The Finnish Institute of Health and Welfare provides bi-annually updated national statistics on CAD and DM patients’ LDL-C levels. That data shows a uniform decline in LDL-C levels towards lower values in both CAD and DM patients of all ages [19,20]. One factor influencing our results may be the mid-study update and tightening of treatment targets [1,3]. This indicates that the tightened treatment targets had been put into use. It would be interesting to follow up on whether a longer observation period after the updated and tightened recommendations would affect the treatment and LDL-C levels of our patients.

Several factors should be considered when aiming to achieve successful treatment with LLM. We observed that patients near the LDL-C value of 3 were less likely to be prescribed with LLM. This might be due to clinical inertia or patients’ own decision [16,21–23]. Maintaining compliance with prescribed long-term medications is a worldwide issue recognised by the World Health Organization [24]. LLMs lower LDL-C more effectively if taken regularly [25]. Dyslipidaemia is also an asymptomatic condition which may affect adherence to effective care [26]. In the twenty first century, the media has familiarised the public with side effects of statins which leads to poorer medication adherence [23,27]. The negative attitude towards the use of statins, which may lead to discontinuation of medication, has been associated with an increased risk of cardiovascular complications [23]. Interestingly, continuity of care has been linked to statins being prescribed more often, as well as better adherence to statin treatment [28–30]. Providing patients with adequate explanations of the importance of LDL-C, ASCVD risk reduction and LLM may enhance medication adherence [23,31,32]. Motivational interview intervention has been linked to better outcomes in medication adherence [33]. These insights regarding LLM should be acknowledged in clinical care.

### Strengths and limitations

There are some limitations and strengths that should be taken into consideration. On the one hand, not knowing the individual target levels for each patient is a limitation in this study. For the same reason, we could not provide data at 36 months on how many patients had achieved their personalised treatment goal. Thus, we were only able to illustrate this issue through baseline LDL-C risk levels. Another limitation is that we do not know whether the patients used the medications prescribed. The source of the LLM information was the existing nurse-verified medication list, yet that does not tell us what the medication adherence in real life was. On the other hand, a strength of the study is that the patients were selected from the general flow of patients in primary health care with common illnesses. Thus, the study mirrors the study question in everyday clinical practice. The dropout rates remained quite low over the 36 months period, and the observation period is relatively long, which are strengths of the study. In the present study, we combined the two original arms of the randomised controlled trial. We considered that justified because no statistically significant difference in primary outcomes (including LDL-C) was found by the non-targeted participatory care plans of the original study design.

## Conclusion

This study provides a perspective on real-word treatment patterns and changes in medications prescribed as the recommendations change over time. During increasing pharmacotherapeutic opportunities, this study suggests that common lipid-lowering medications are associated with decreasing or maintaining lower LDL-C levels among primary health care patients with chronic ASCVDs. Patients who were achieving the lowest LDL-C levels were prescribed either simvastatin, atorvastatin, or rosuvastatin and/or ezetimibe. The more lipid-lowering medications are prescribed, the more likely it is to achieve lower LDL-C level. A greater decrease was observed among patients with higher baseline LDL-C values. Despite the achieved decrease in LDL-C, this study, conducted in everyday clinical practice suggests that the gap remains between guideline-recommended treatment targets and achieved LDL-C levels despite the availability of pharmacological opportunities to enhance treatment.

## Data Availability

The data of the current study are not publicly available in order to protect individual privacy but are available from the corresponding author upon reasonable request.

## References

[1] Working Group Set Up by the Finnish Medical Society Duodecim, Finnish Society of Internal Medicine. Dyslipidaemias. Current Care Guideline [Internet]; 2022 [cited 2026 Apr 22]. Available from: https://www.kaypahoito.fi/hoi50025

[2] WHO. World health statistics; 2024 [Internet]. [cited 2026 Apr 22]. Available from: https://iris.who.int/server/api/core/bitstreams/74b12494-f213-4b5b-9533-18442147e1fb/content

[3] 2019 ESC/EAS. Guidelines for the management of dyslipidaemias: lipid modification to reduce cardiovascular risk. | European Heart Journal | Oxford Academic [Internet]. [cited 2026 Apr 22]. Available from: https://academic.oup.com/eurheartj/article/41/1/111/5556353?login=false#207091830 10.1093/eurheartj/ehz455 31504418

[4] Pirillo A, Casula M, Olmastroni E, et al. Global epidemiology of dyslipidaemias. Nat Rev Cardiol. 2021;18(10):689–700. doi: 10.1038/s41569-021-00541-4. 33833450

[5] Mach F, Koskinas KC, Roeters van Lennep JE, et al. 2025 Focused update of the 2019 ESC/EAS guidelines for the management of dyslipidaemias: developed by the task force for the management of dyslipidaemias of the European Society of Cardiology (ESC) and the European Atherosclerosis Society (EAS). Eur Heart J. 2025;46(42):4359–4378. doi: 10.1093/eurheartj/ehaf190. 40878289

[6] Jones JE, Tang KS, Barseghian A, et al. Evolution of more aggressive LDL-cholesterol targets and therapies for cardiovascular disease prevention. J Clin Med. 2023;12(23):7432. doi: 10.3390/jcm12237432. 38068483 PMC10707224

[7] Baigent C, Blackwell L, Emberson J, et al. Efficacy and safety of more intensive lowering of LDL cholesterol: a meta-analysis of data from 170,000 participants in 26 randomised trials. Lancet. 2010;376(9753):1670–1681. doi: 10.1016/S0140-6736(10)61350-5. 21067804 PMC2988224

[8] Komnianou A, Kyriakoulis KG, Menti A, et al. Cardiovascular risk assessment and lipid-lowering therapy recommendations in primary prevention. J Clin Med. 2025;14(7):2220. doi: 10.3390/jcm14072220. 40217673 PMC11989271

[9] Gouni-Berthold I, Schaper F, Schatz U, et al. Low-density lipoprotein cholesterol goal attainment in Germany: results from the DA VINCI study. Atheroscler Plus. 2022;50:10–16. doi: 10.1016/j.athplu.2022.07.024. 36643801 PMC9833225

[10] Ray KK, Haq I, Bilitou A, et al. Treatment gaps in the implementation of LDL cholesterol control among high- and very high-risk patients in Europe between 2020 and 2021: the multinational observational SANTORINI study. Lancet Reg Health Eur. 2023;29. doi: 10.1016/j.lanepe.2023.100624. PMC10119631 37090089

[11] Tahkola A, Korhonen P, Kautiainen H, et al. Lifetime risk assessment in cholesterol management among hypertensive patients: observational cross-sectional study based on electronic health record data. BMC Fam Pract. 2020;21(1):62. doi: 10.1186/s12875-020-01138-5. 32290820 PMC7155316

[12] Tusa N, Kautiainen H, Elfving P, et al. Randomized controlled study of the impact of a participatory patient care plan among primary care patients with common chronic diseases: a one-year follow-up study. BMC Health Serv Res. 2021;21(1):715. doi: 10.1186/s12913-021-06716-6. 34284783 PMC8293542

[13] Tusa N, Mikkonen U, Kautiainen H, et al. Participatory care plan for primary care patients with long-term diseases: results after a 36-month follow-up of a randomized controlled trial. BMC Health Serv Res. 2025;25(1):536. doi: 10.1186/s12913-025-12660-6. 40217248 PMC11992695

[14] Bush K, Kivlahan DR, McDonell MB, et al. The AUDIT alcohol consumption questions (AUDIT-C): an effective brief screening test for problem drinking. Ambulatory Care Quality Improvement Project (ACQUIP). Alcohol Use Disorders Identification Test. Arch Intern Med. 1998;158(16):1789–1795. doi: 10.1001/archinte.158.16.1789. 9738608

[15] Kasari D. Effect of exercise and fitness on serum lipids in college women. University of Montana; 1976.

[16] Nasir K, Salami JA. Therapeutic inertia in lipid-lowering treatment intensification: digital tools and performance management to the rescue? Circ Cardiovasc Qual Outcomes. 2022;15(12):e009399. doi: 10.1161/CIRCOUTCOMES.122.009399. 36256462

[17] Bilitou A, Were J, Farrer A, et al. Prevalence and patient outcomes of adult primary hypercholesterolemia and dyslipidemia in the UK: longitudinal retrospective study using a primary care dataset from 2009 to 2019. Clinicoecon Outcomes Res. 2022;14:189–203. doi: 10.2147/CEOR.S347085. 35411162 PMC8994561

[18] Magnussen C, Ojeda FM, Leong DP, et al. Global effect of modifiable risk factors on cardiovascular disease and mortality. N Engl J Med. 2023;389(14):1273–1285. doi: 10.1056/NEJMoa2206916. 37632466 PMC10589462

[19] Finnish Institute for Health and Welfare. Nationwide cardiovascular registry of Finland. [Internet]. [cited 2026 Apr 22]. Available from: https://www.thl.fi/kansallisten-laaturekisterien-raportit/sydanrekisteri/sydanrekisteri_kokosuomi.html

[20] Finnish Institute for Health and Welfare. Reports from the nationwide quality registry [Internet]. 2026 [cited 2026 Apr 22]. Available from: https://thl.fi/aiheet/sote-palvelujen-johtaminen/arviointi-ja-seuranta/sote-tietopohja/terveydenhuollon-kansalliset-laaturekisterit/kansallisten-laaturekisterien-raportit

[21] García Díaz E, Ramírez Medina D, Morera Porras ÓM, et al. Determinants of inertia with lipid-lowering treatment in patients with type 2 diabetes mellitus. Endocrinol Diabetes Nutr (Engl Ed). 2019;66(4):223–231. doi: 10.1016/j.endinu.2018.08.014. 30541682

[22] Desai NR, Farbaniec M, Karalis DG. Nonadherence to lipid-lowering therapy and strategies to improve adherence in patients with atherosclerotic cardiovascular disease. Clin Cardiol. 2023;46(1):13–21. doi: 10.1002/clc.23935. 36267039 PMC9849440

[23] Mehta A. Non adherence to lipid-lowering therapy and strategies to improve adherence. Indian Heart J. 2024;76(Suppl 1):S138–S140. doi: 10.1016/j.ihj.2024.01.006. 38211773 PMC11019324

[24] Failure to take prescribed medicine for chronic diseases is a massive, world-wide problem [Internet]. [cited 2026 Apr 22]. Available from: https://www.who.int/news/item/01-07-2003-failure-to-take-prescribed-medicine-for-chronic-diseases-is-a-massive-world-wide-problem

[25] Li J, Mudiganti S, Husby H, et al. Factors and outcomes associated with adherence to statins among patients with newly diagnosed cardiovascular disease. Am J Prev Cardiol. 2025;23:101028. doi: 10.1016/j.ajpc.2025.101028. 40585340 PMC12206012

[26] Ju A, Hanson CS, Banks E, et al. Patient beliefs and attitudes to taking statins: systematic review of qualitative studies. Br J Gen Pract. 2018;68(671):e408–e419. doi: 10.3399/bjgp18X696365. 29784867 PMC6002012

[27] Kriegbaum M, Liisberg KB, Wallach-Kildemoes H. Pattern of statin use changes following media coverage of its side effects. Patient Prefer Adherence. 2017;11:1151–1157. doi: 10.2147/PPA.S133168. 28744105 PMC5513880

[28] Tammes P, Payne RA, Salisbury C. Association between continuity of primary care and both prescribing and adherence of common cardiovascular medications: a cohort study among patients in England. BMJ Open. 2022;12(9):e063282. doi: 10.1136/bmjopen-2022-063282. PMC9472141 36100300

[29] Youens D, Doust J, Robinson S, et al. Regularity and continuity of GP contacts and use of statins amongst people at risk of cardiovascular events. J Gen Intern Med. 2021;36(6):1656–1665. doi: 10.1007/s11606-021-06638-3. 33655384 PMC8175539

[30] Warren JR, Falster MO, Tran B, et al. Association of continuity of primary care and statin adherence. PLoS One. 2015;10(10):e0140008. doi: 10.1371/journal.pone.0140008. 26448561 PMC4598138

[31] Türkmen D, Liang X, Masoli JAH, et al. Understanding the causes and consequences of low statin adherence: evidence from UK Biobank primary care data. BMC Med. 2025;23(1):436. doi: 10.1186/s12916-025-04228-2. 40696379 PMC12285031

[32] Kök M, Bostan F, Yacan Kök A. Perceptions and knowledge of cholesterol and lipid-lowering medications among treatment-naive individuals: a cross-sectional study. Patient Prefer Adherence. 2025;19:1411–1422. doi: 10.2147/PPA.S511454. 40386799 PMC12085121

[33] Abughosh SM, Vadhariya A, Johnson ML, et al. Enhancing statin adherence using a motivational interviewing intervention and past adherence trajectories in patients with suboptimal adherence. J Manag Care Spec Pharm. 2019;25(10):1053–1062. doi: 10.18553/jmcp.2019.25.10.1053. 31556824 PMC10398332

